# Ergothioneine Ameliorates Alcoholic Fatty Liver Disease: A Dual Strategy of Accelerated Ethanol Elimination and Reducing Oxidative Stress

**DOI:** 10.1002/jbt.70899

**Published:** 2026-05-12

**Authors:** Wei Ding, Juan Cao, Cong Guo, Xu Li, Wei Liu, Guohua Xiao

**Affiliations:** ^1^ Gene III Biotechnology Co. Ltd. Nanjing Jiangsu Province China

**Keywords:** ergothioneine, hepatic steatosis, hepatoprotection, lipid metabolism, NIAAA mouse model

## Abstract

Alcoholic fatty liver disease (AFLD) is a progressive hepatic pathology triggered by chronic ethanol consumption, serving as the initial stage of severe liver injury. Currently, there are no Food and Drug Administration (FDA)‐approved pharmacological interventions that specifically target alcohol‐induced hepatic steatosis or prevent disease progression, highlighting an urgent need for effective preventive strategies. This study evaluated the preventive efficacy and underlying mechanisms of Ergothioneine (EGT) in a clinically relevant preclinical model. C57BL/6 mice were randomized into five groups: a Control group, an alcoholic fatty liver Model group, a Positive control group treated with Silybin (100 mg/kg), and three EGT treatment groups (10, 30, and 50 mg/kg). The National Institute on Alcohol Abuse and Alcoholism (NIAAA) mouse model was utilized to induce alcoholic fatty liver. Various biochemical, histological, and molecular markers were assessed to evaluate liver damage, alcohol metabolism, lipid profiles, oxidative stress, and inflammation. EGT treatment significantly ameliorated hepatic steatosis and necrosis, as confirmed by Hematoxylin and Eosin (H&E) and Oil Red O staining. Notably, EGT accelerated alcohol clearance, reducing serum ethanol levels by up to 54.4% in a dose‐dependent manner. Furthermore, EGT restored liver function markers, including alanine aminotransferase (ALT), aspartate aminotransferase (AST), and gamma‐glutamyl transferase (GGT), and corrected dyslipidemia by lowering triglycerides (TG), total cholesterol (TC), and low‐density lipoprotein cholesterol (LDL‐C) while elevating high‐density lipoprotein cholesterol (HDL‐C). Mechanistically, EGT suppressed pro‐inflammatory cytokines (Interleukin‐6 [IL‐6], Interleukin‐1 beta [IL‐1β]) and mitigated oxidative stress by reducing malondialdehyde (MDA) accumulation and restoring superoxide dismutase (SOD) and glutathione peroxidase (GSH‐Px) activities. Ergothioneine prevents alcoholic liver injury through a dual mechanism: accelerating ethanol metabolism and enhancing hepatocyte antioxidative and anti‐inflammatory defenses. These findings position EGT as a promising therapeutic candidate for AFLD management.

## Graphical abstract

Ergothioneine (EGT) ameliorates alcoholic fatty liver disease via a dual mechanism: accelerating ethanol clearance by protecting ADH/ALDH activity (serum EtOH decreased 54.4%), and reducing oxidative stress/inflammation via antioxidant restoration (GSH‐Px increased 198.2%) and NF‐κB suppression (IL‐1β decreased 67.1%), collectively reversing hepatic steatosis and injury.

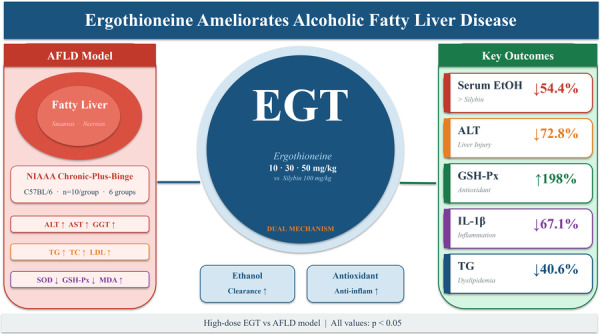

## 1 Introduction

Alcoholic fatty liver disease represents the earliest and most prevalent stage of alcoholic liver disease, characterized by excessive triglyceride accumulation in the liver as a direct consequence of chronic ethanol abuse [1]. The pathogenesis of AFLD involves complex mechanisms driven primarily by oxidative stress and disruptions of normal lipid metabolism, which can result in liver damage [2]. Specifically, ethanol metabolism induces the overexpression of cytochrome P450 2E1 and activates nicotinamide adenine dinucleotide phosphate (NADPH) oxidase, leading to excessive production of reactive oxygen species and subsequent depletion of glutathione [3, 4]. Furthermore, alcohol‐induced gut dysbiosis increases intestinal permeability, allowing endotoxins to reach the liver and activate Kupffer cells, thereby triggering an inflammatory cascade dominated by cytokines such as interleukin‐6 (IL‐6), interleukin‐1 beta (IL‐1β), and tumor necrosis factor‐alpha (TNF‐α) [5]. This imbalance precipitates hepatic oxidative stress, triggering lipid peroxidation, mitochondrial dysfunction, and inflammatory responses that ultimately drive hepatocyte apoptosis and necrosis [6]. While simple steatosis is often considered reversible, it sensitizes the liver to “second hits” such as oxidative stress and inflammation, potentially progressing to steatohepatitis, fibrosis, and cirrhosis if left unchecked.

The global burden of AFLD is substantial, with estimates indicating that alcohol‐related harm accounts for over 3 million deaths annually worldwide and contributes to more than 200 health conditions [7]. The current standard of care relies on abstinence [8, 9] and nutritional support, with pharmacological agents like Silybin used as supportive therapy. However, traditional antioxidants often lack specific transport mechanisms to achieve high intracellular concentrations in mitochondria, the primary site of reactive oxygen species (ROS) Generation.

Ergothioneine (EGT), a naturally occurring thiol‐histidine derivative, has emerged as a potent dietary antioxidant distinguished by its unique transport system and capacity to mitigate cellular oxidative damage and inflammation [10]. Recent studies have highlighted EGT's potential in metabolic dysfunction‐associated steatotic liver disease (MASLD) via autophagy enhancement and ferroptosis inhibition [10, 11]. Despite its established antioxidant properties, the specific efficacy of EGT in preventing alcoholic fatty liver, particularly within the rigorous National Institute on Alcohol Abuse and Alcoholism (NIAAA) mouse model framework, has not been thoroughly investigated.

Therefore, this study aims to evaluate the preventive effects of EGT on ethanol‐induced hepatic steatosis and injury by comparing its efficacy against Silybin, a standard hepatoprotective agent, in a well‐established mouse model of AFLD. Consequently, elucidating the mechanisms by which EGT modulates alcohol metabolism, lipid profiles, and inflammatory cytokines may provide critical insights for developing novel therapeutic strategies for AFLD.

## 2 Materials and Methods

### 2.1 Chemicals and Reagents

Ergothioneine (>99.99% purity) was supplied by Jiangsu Gene III Biotechnology Co. Ltd. (Nanjing, China). Silybin (> 98.76%) was obtained from Shandong Sikejie Biotechnology Co. Ltd. The Lieber‐DeCarli ethanol and control liquid diets were purchased from Xietong Bio (China). Diagnostic kits for ALT, AST, GGT, TG, TC, LDL‐C, and HDL‐C were obtained from Nanjing Jiancheng Bioengineering Institute (China). ELISA kits for IL‐1β, TNF‐α, and IL‐6 were purchased from Abclonal (China). Kits for GSH‐Px, MDA, and Ethanol detection were sourced from Beyotime (China), and the SOD kit was obtained from Addison (China).

### 2.2 Animals and Experimental

Design Specific Pathogen Free (SPF) male C57BL/6 mice (8 weeks old) were obtained from Jiangsu Huachuang Xinnuo Pharmaceutical Technology Co. Ltd. Mice were housed under standard conditions (24°C–28°C, 12 h light/dark cycle). All animal procedures were approved by the Animal Ethics Committee (Approval No.: SYXK‐2023‐0019).

The NIAAA chronic‐plus‐binge model was established as previously described. After acclimatization, mice were randomized into six groups (*n* = 10/group): (1) Control, (2) Model (ETOH), (3) Positive Control (Silybin 100 mg/kg), and (4–6) EGT treatment groups (10, 30, and 50 mg/kg). Pretreatment with EGT or Silybin was administered intragastrical for 10 days. Subsequently, ethanol groups were fed the Lieber‐DeCarli ethanol liquid diet (5% ethanol) for 10 days, while the Control group received an isocaloric control diet. On day 11, ethanol groups received a single binge of ethanol (5 g/kg), whereas controls received maltodextrin. Mice were sacrificed 9 h post‐binge to collect serum and liver tissues.

### 2.3 Histopathological Analysis

Liver tissues were fixed in 4% paraformaldehyde, embedded in paraffin, and sectioned (5 μm) for Hematoxylin and Eosin (H&E) staining. For lipid droplet visualization, frozen liver sections embedded in OCT compound were subjected to Oil Red O (ORO) staining. Images were captured using a light microscope.

### 2.4 Biochemical Analysis

Serum samples were separated by centrifugation at 4000 rpm for 10 min at 4°C. The levels of liver injury markers (ALT, AST, GGT), lipid profiles (TG, TC, LDL‐C, HDL‐C), and serum ethanol concentration were quantified using commercial assay kits strictly according to the manufacturers' instructions. Absorbance was measured using a microplate reader.

### 2.5 Inflammatory and Oxidative Stress Assessment

Serum levels of inflammatory cytokines (IL‐6, IL‐1β, TNF‐α) were determined using enzyme‐linked immunosorbent assay (ELISA) kits following the standard protocols. Oxidative stress markers, including MDA content and the activities of SOD and GSH‐Px, were assessed using specific colorimetric assay kits according to the manufacturers' procedures.

### 2.6 Statistical Analysis

Data are expressed as mean ± standard deviation (SD). Statistical differences were analyzed using GraphPad Prism software via one‐way analysis of variance (ANOVA) followed by Bonferroni's post hoc test. A *p*‐value < 0.05 was considered statistically significant.

## 3 Results

### 3.1 Effect of EGT on Liver Histology in NIAAA Mice

**Figure 1:**
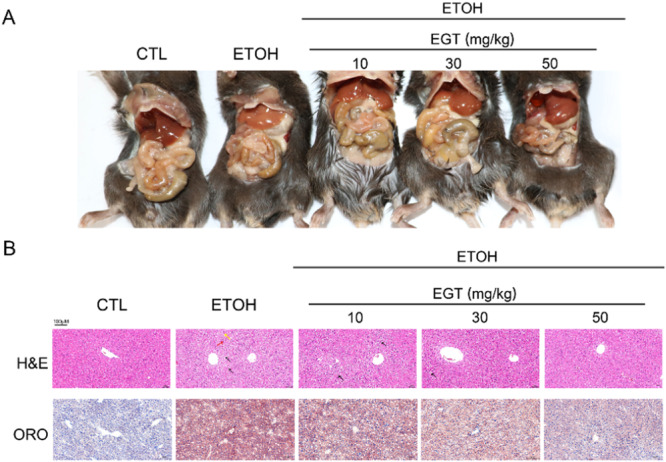
Effect of EGT on Liver Tissue of NIAAA. Mice A mouse alcoholic fatty liver model was established to verify the effect of EGT on mouse liver tissue. (A) Mouse anatomy diagram. (B) Representative photos of H&E sections and Oil Red O staining of mouse liver tissue (200×).

To investigate the protective effect of EGT, we established an NIAAA mouse model. Morphologically, EGT treatment improved the appearance of fatty livers (Figure 1A). Histopathological analysis (H&E) revealed that the Control group exhibited normal hepatic cord structure with orderly arranged hepatocytes. In contrast, the Model group showed varying sizes of lipid droplets (black arrows), inflammatory cell infiltration (yellow arrows), and hepatocyte necrosis (red arrows). Compared to the Model group, mice treated with Gene III EGT showed more regular hepatocyte morphology, significantly reduced lipid droplets, and alleviated inflammatory infiltration and pathological injury (Figure 1B). Consistently, Oil Red O staining demonstrated massive red lipid droplet accumulation in the Model group, which was significantly attenuated by EGT administration (Figure 1B). These results confirm that EGT alleviates alcoholic fatty liver in mice.

### 3.2 Effect of EGT on Serum Ethanol Levels

**Figure 2:**
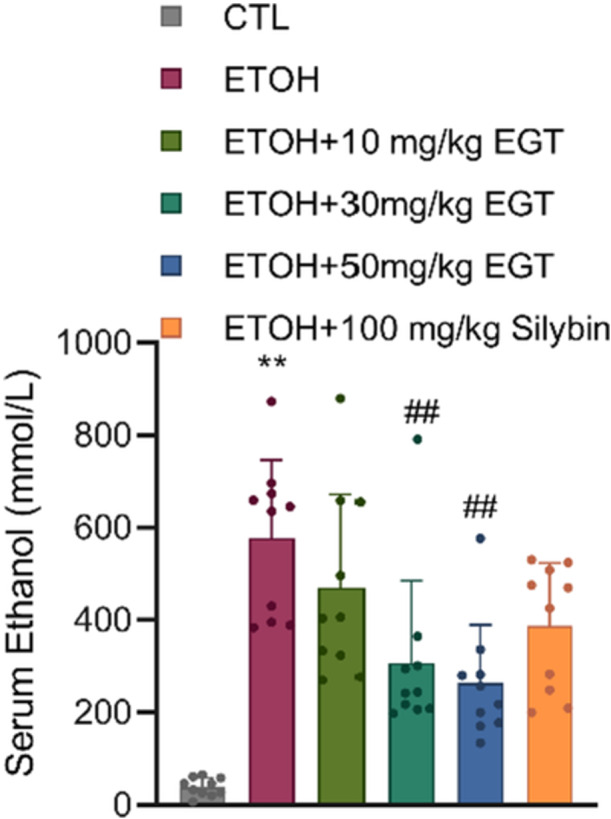
Effect of EGT on Serum Alcohol Content in NIAAA Mice. Alcohol content in mouse serum. *n* = 10, ** *p* < 0.01 v.s. CTL group, ## *p* < 0.01 v.s. ETOH group, data are expressed as Mean ± SD.

As shown in Figure 2, serum ethanol levels were significantly elevated in the Model group compared to the Control group. Gene III EGT administration reduced serum ethanol levels by 20.4%, 45.2%, and 54.4% in the low, medium, and high‐dose groups, respectively, compared to the Model group, exhibiting a dose‐dependent trend. This suggests that EGT promotes alcohol metabolism, thereby lowering serum alcohol content. And this magnitude of reduction significantly exceeded that of Silybin (~30% reduction), suggesting that EGT may actively enhance the enzymatic clearance of ethanol or protect metabolic enzymes from inactivation.

### 3.3 Effect of EGT on Liver Injury Markers

**Figure 3:**
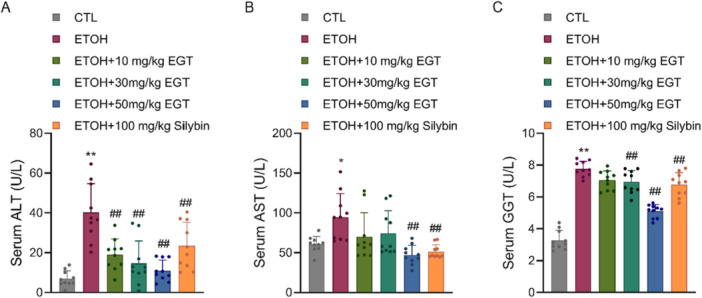
Effect of EGT on Liver Injury Indicators in NIAAA Mice. (A) Mouse serum ALT content. (B) Mouse serum AST content. (C) Mouse serum GGT content. *n* = 10, *p < 0.05, **p < 0.01 v.s. CTL group, ## *p* < 0.01 v.s. ETOH group, data are expressed as Mean ± SD.

Compared to the Control group, the Model group showed significantly increased serum levels of ALT, AST, and GGT. EGT treatment significantly reduced these markers. Specifically, AST levels decreased by 25.8%, 21.5%, and 50.2% (Figure 3A); ALT levels decreased by 52.6%, 63.7%, and 72.8% (Figure 3B); and GGT levels decreased by 9%, 10.4%, and 33% (Figure 3C) compared to the Model group. These findings confirm the hepatoprotective effect of EGT.

### 3.4 Effect of EGT on Serum Lipid Profiles

**Figure 4:**
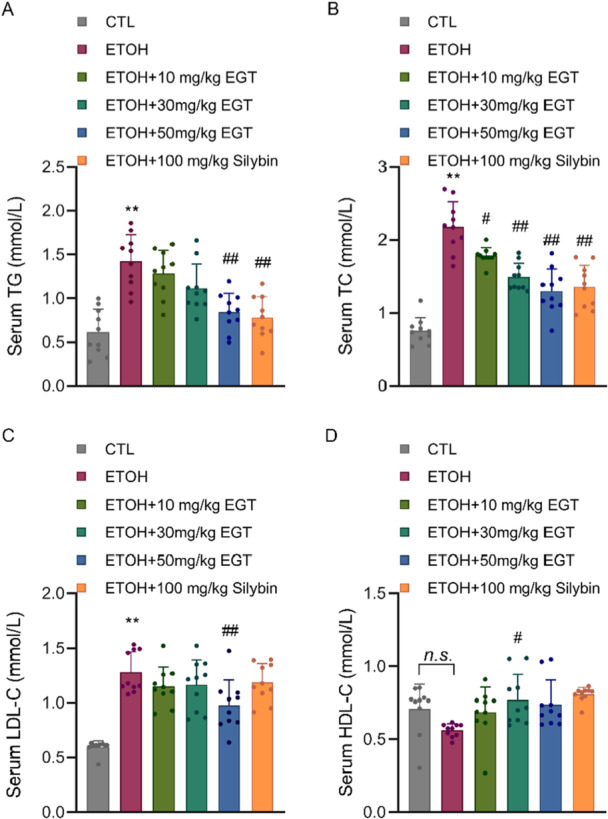
Effect of EGT on Blood Lipid Content in NIAAA Mice. (A) Mouse serum TG content. (B) Mouse serum TC content. (C) Mouse serum LDL‐C content. (D) Mouse serum HDL‐C content. *n* = 10, **p < 0.01 v.s. CTL group, # *p* < 0.05, ## *p* < 0.01 v.s. ETOH group, n.s.: no significance, data are expressed as Mean ± SD.

Regarding lipid biochemical indicators, serum TG and TC levels were significantly elevated in the Model group compared to the Control group. EGT treatment reduced TG levels by 10.6%, 20.3%, and 40.6%, and TC levels by 17.6%, 31.4%, and 40.4%, respectively (Figure 4A, B). Additionally, LDL‐C levels, which were elevated in the Model group, decreased by 9.3%, 8.1%, and 22.9% following EGT treatment (Figure 4C). Conversely, HDL‐C levels, which showed a decreasing trend in the Model group, increased by 20.96%, 35%, and 31.4% with EGT treatment (Figure 4D). This indicates that EGT promotes the restoration of normal serum lipid profiles.

### 3.5 Effect of EGT on Oxidative Stress Indices

**Figure 5:**
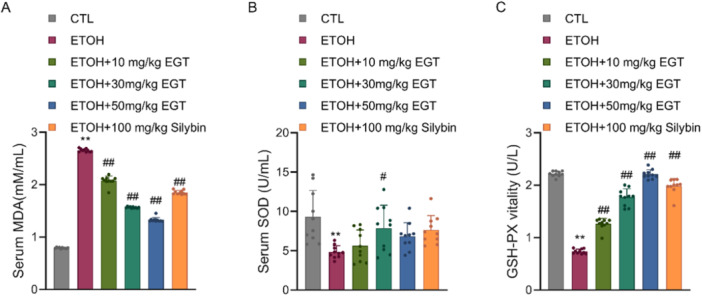
Effect of EGT on Oxidative Stress Indicators in NIAAA Mice. (A) Mouse serum MDA content. (B) Mouse serum SOD content. (C) Mouse serum GSH‐PX content. *n* = 10, ** *p* < 0.01 v.s. CTL group, # *p* < 0.05, ## *p* < 0.01 v.s. ETOH group, data are expressed as Mean ± SD.

Regarding oxidative stress, serum MDA levels were significantly increased in the Model group. EGT treatment reduced MDA levels by 21.5%, 41%, and 50.2% in a concentration‐dependent manner (Figure 5A). Furthermore, the activities of antioxidant enzymes SOD and GSH‐Px were significantly decreased in the Model group. Compared to the Model group, EGT treatment increased SOD levels by 16.4%, 62.3%, and 46.7% (Figure 5B), and GSH‐Px activity by 71.4%, 140.2%, and 198.2% (Figure 5C). These results suggest that EGT improves the oxidative stress status in mice.

Interestingly, while the high‐dose EGT (50 mg/kg) significantly elevated SOD activity compared to the model group (+46.7%), the magnitude of this increase was slightly lower than that of the medium‐dose group (+62.3%). This observation may be attributed to a homeostatic feedback mechanism. Given that EGT itself is a potent direct scavenger of reactive oxygen species (ROS), the high intracellular concentration of EGT might have sufficiently reduced the basal oxidative stress signal required to induce maximal SOD expression. Furthermore, considering the continuous and robust surge in GSH‐Px activity at the high dose (+198.2%), this pattern suggests a synergistic shift in the antioxidant defense strategy: prioritizing the clearance of downstream peroxides (via GSH‐Px) to prevent the accumulation of hydrogen peroxide produced by SOD, thereby optimizing the overall detoxification efficiency.

### 3.6 Effect of EGT on Serum Pro‐Inflammatory Cytokines

**Figure 6:**
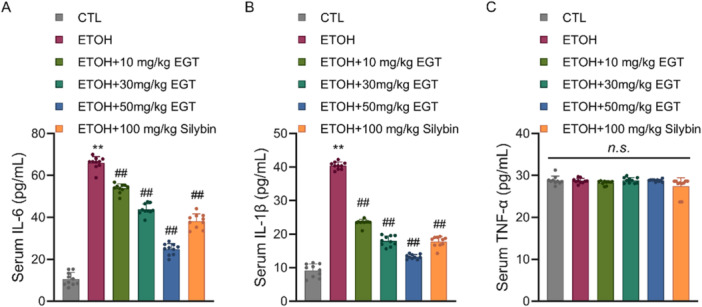
Effect of EGT on Serum pro‐inflammatory cytokines in NIAAA Mice. (A) Mouse serum IL‐6 content. (B) Mouse serum IL‐1β content. (C) Mouse serum TNF‐α content. n = 10, **p < 0.01 v.s. CTL group, ## *p* < 0.01 v.s. ETOH group, n.s.: no significance, data are expressed as Mean ± SD.

Cytokines in terms of inflammation, the Model group exhibited significantly elevated levels of pro‐inflammatory cytokines IL‐6 and IL‐1β compared to the Control group. EGT treatment significantly lowered serum IL‐6 levels by 17.5%, 33%, and 60.5%, and IL‐1β levels by 42.1%, 55.6%, and 67.1%, in a concentration‐dependent manner (Figure 6A, B). Interestingly, TNF‐α levels did not show significant variance at the 9‐h post‐binge time point, likely due to its transient peak occurring earlier in the inflammatory cascade (Figure 6C). This suggests that EGT alleviates the inflammatory state in mice.

## 4 Discussion

This study systematically evaluated the preventive efficacy of EGT on alcoholic fatty liver disease (AFLD) using the NIAAA chronic‐plus‐binge mouse model. Our findings demonstrate that EGT not only significantly ameliorates hepatic pathology, reduces liver injury markers, and restores lipid homeostasis but also reveals a unique “dual‐defense mechanism”: preventing liver injury through the synergistic action of accelerating ethanol metabolic clearance and enhancing endogenous hepatocellular antioxidant/anti‐inflammatory defenses.

The most striking finding of this study is the capacity of EGT to accelerate alcohol clearance. High‐dose EGT reduced serum ethanol levels by 54.4%, significantly outperforming the standard agent Silybin. We hypothesize that this effect may be attributed to the protection of alcohol dehydrogenase (ADH) and aldehyde dehydrogenase (ALDH) from oxidative inactivation [12]. As a stable thione‐thiol antioxidant, EGT may maintain the structural integrity of critical thiol groups within these metabolic enzymes, ensuring efficient alcohol metabolism and thereby reducing the sustained toxicity of ethanol at its source [13].

Regarding oxidative stress, EGT treatment significantly reduced lipid peroxidation (MDA) and restored antioxidant enzyme activities in a distinct synergistic pattern. While SOD activity in the high‐dose EGT group was significantly elevated compared to the Model group, the magnitude of induction was slightly lower than in the medium‐dose group, showing a non‐linear trend. In contrast, GSH‐Px activity exhibited a continuous and robust dose‐dependent surge (increasing nearly twofold). This phenomenon likely reflects a homeostatic feedback regulation of the antioxidant system: the potent direct radical‐scavenging ability of high‐concentration EGT may reduce the oxidative stress threshold required to induce SOD [14]. Simultaneously, the liver prioritizes a substantial upregulation of the downstream detoxifying enzyme (GSH‐Px) to ensure the rapid clearance of hydrogen peroxide generated by SOD. This “demand‐based” enzyme regulation highlights the intelligent characteristic of EGT in maintaining cellular redox balance.

Furthermore, EGT effectively blocked the inflammatory cascade by inhibiting the release of pro‐inflammatory cytokines IL‐6 and IL‐1β, potentially via the suppression of the NF‐κB signaling pathway [15]. In terms of lipid metabolism, EGT not only improved the lipid profile but also significantly elevated HDL‐C levels. While our current findings robustly demonstrate the potent antioxidant capacity of EGT through the significant reduction of MDA and the restoration of SOD and GSH‐Px activities, we acknowledge certain limitations in our oxidative stress evaluation panel. Oxidative liver damage is a highly complex process involving not only lipid peroxidation but also protein oxidation and DNA damage. For a more detailed and comprehensive insight into oxidative liver damage, future studies should incorporate additional biomarkers, such as Advanced Oxidation Protein Products (AOPP) to assess protein damage, and 8‐hydroxy‐2′‐deoxyguanosine (8‐OHdG) to evaluate oxidative DNA damage [16]. Similarly, while GSH‐Px activity was measured, directly assessing the levels of reduced glutathione (GSH) would provide a more direct indicator of the intracellular antioxidant protection capacity, as GSH depletion is a hallmark of alcohol‐induced oxidative stress [17]. Nevertheless, the substantial upregulation of downstream detoxifying enzymes observed in our current NIAAA model provides compelling foundational evidence of EGT's protective mechanism against alcohol‐induced oxidative stress.

Given that EGT is specifically accumulated in mitochondria via the OCTN1 transporter [10, 18], it likely maintains fatty acid oxidation efficiency by protecting mitochondrial function and may also facilitate lipophagy to accelerate lipid droplet degradation [11].

In conclusion, EGT effectively prevents alcoholic liver injury through a dual mechanism of “metabolic enhancement” and “cytoprotection.” These findings provide a solid scientific basis for developing EGT as a novel functional food or therapeutic agent for the prevention of AFLD.

## Author Contributions

**Wei Ding:** conceptualization, supervision, project administration. **Juan Cao:** conceptualization, project administration, supervision, data curation. **Cong Guo:** investigation, validation, methodology, formal analysis, supervision, project administration. **Xu Li:** investigation, validation, methodology, formal analysis, project administration, supervision, data curation. **Wei Liu:** writing – original draft, writing – review and editing, investigation, validation, formal analysis. **Guohua Xiao:** conceptualization, data curation, supervision, methodology, project administration.

## Funding

The authors have nothing to report.

## Conflicts of Interest

The authors declare no conflicts of interest.

## Data Availability

The data presented in this study are available on request from the corresponding author. The data are not publicly available due to privacy restrictions.
